# Impetigo Surgical Site Infection Following Open Reduction and Internal Fixation of a Patella Fracture

**DOI:** 10.7759/cureus.52259

**Published:** 2024-01-14

**Authors:** Westin M Yu, Tristan M Palmer, Drew Miller, Shawn Storm

**Affiliations:** 1 Surgery, Lake Erie College of Osteopathic Medicine, Erie, USA; 2 Orthopaedic Surgery, LECOM Health Millcreek Community Hospital, Erie, USA

**Keywords:** staph aures, surgical fixation of patella fracture, open reduction and internal fixation with a plate (orif with plate), surgical site infection(ssi), impetigo, patella fracture

## Abstract

Impetigo is a pediatric skin infection characterized by the presence of pathognomonic "honey-crusted" lesions caused by either *Staphylococcus aureus *or *Streptococcus pyogenes*. The diagnosis of impetigo is largely based on clinical judgment, confirmatory skin cultures, and Gram staining. Surgical site infections following patellar surgery are a relatively uncommon occurrence, with the most common causative organisms being *Staphylococcus aureus*, *Streptococcus pyogenes, Pseudomonas aeruginosa,* and other gram-negative bacilli. Surgical site infections have a range of risk factors that largely depend on patient characteristics and surgical logistics. We report the first documented case in the literature of a surgical impetigo infection with diagnostic skin lesions following open reduction and internal fixation of a patellar fracture in a 24-year-old female.

## Introduction

Patellar fractures account for approximately one percent of all fractures, with an increasing prevalence with age and a discrepancy in genetic sex predominance [1,2]. Treatment can vary from conservative management to surgery, with typical surgical indications including a disruption in the extensor mechanism, open fractures, and an articular stepoff of two millimeters or greater [3]. Surgical techniques can differ between surgeons and the morphology of the fracture, with up to 85% of patients and surgeons reporting a positive surgical outcome regarding quality of life [3]. Surgical fixation does not come without risk, as there is a reported 2-6% rate of surgical site infection and up to a 33% re-operation rate [4,5].

Impetigo is divided into a bullous and nonbullous type, with the nonbullous type comprising 70% of all cases [6]. Bullous impetigo is almost exclusively caused by Staphylococcus aureus, while the nonbullous type is most commonly caused by Staphylococcus aureus; however, Streptococcus pyogenes is also a known culprit [6]. Both types of impetigo are considered primarily pediatric infections, as it is the most common bacterial skin infection in children two to five years of age [6]. Although adults are not the classical demographic, impetigo can occur at any age. Surgical site infections can vary widely based on a variety of factors, including patient comorbidities, associated trauma, and operation type, with select operations reporting up to a 20% incidence of infection [7]. Staphylococcus aureus, Streptococcus pyogenes, Pseudomonas aeruginosa, and other gram-negative bacilli are the most commonly identified causative pathogens. We report the first documented case within the literature of an adult impetigo surgical site infection with the characteristic "honey-crusted lesions" after a patellar fracture requiring open reduction and internal fixation.

## Case presentation

A 24-year-old female without significant past medical history or known allergies presented to the orthopedic offices for treatment and management of a right patellar fracture previously diagnosed at an outside urgent care facility. Eleven days prior to her presentation to the clinic, the patient had sustained an injury to her right knee while at work, which involved her right lower extremity being pinned between a truck and a work pallet. She presented to an urgent care facility at the time of injury, where physical examination and X-rays of the right knee demonstrated a closed, displaced, intra-articular patellar fracture. She was treated with a knee immobilizer and crutches and instructed on non-weight-bearing status.

During a follow-up examination in the orthopedic clinic, the patient's injury of a closed, right, intra-articular patellar fracture was confirmed. In addition to her injury, she was found to have an incompetent extensor mechanism, as she was unable to perform an active straight leg raise. Due to the nature of her injury and the above-mentioned physical exam findings, the patient was offered treatment in the form of surgical open reduction and internal fixation. After a discussion of likely outcomes following operative and non-operative treatment, the patient agreed and consented to surgical fixation.

**Figure 1:**
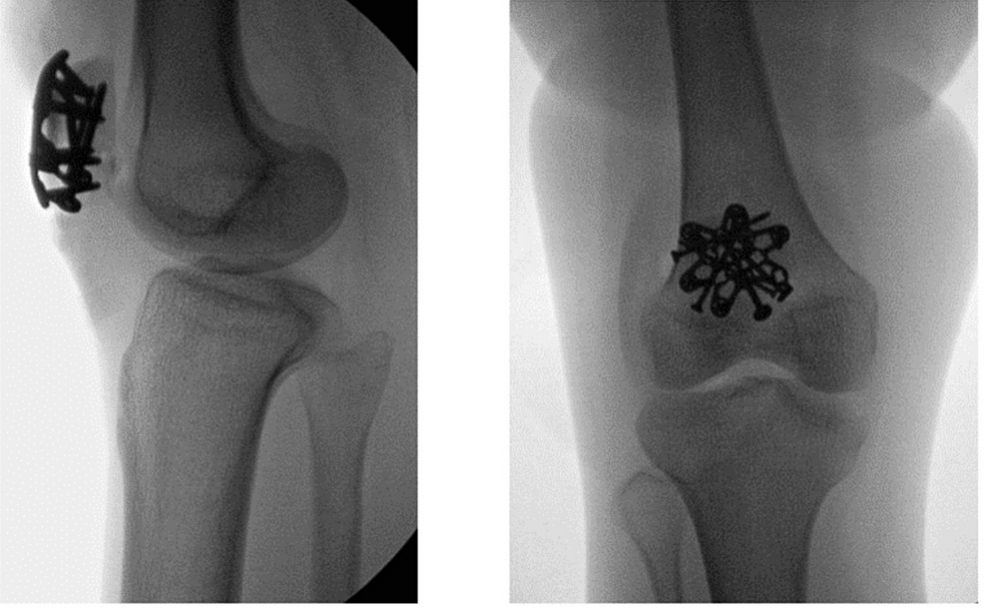
Intra-operative anterior-posterior and lateral images of the patient's right knee following open reduction and internal fixation of a patellar fracture

The patient was taken to surgery on the twelfth day following her injury. Due to the patient's body weight of 43kg, one gram of cefazolin was given intravenously before the inflation of the tourniquet and incision. A 10 cm midline incision was used to access the fracture. The retinaculum of the knee was found to be torn, and no additional arthrotomy was performed. The fracture was reduced with bone clamps. The fracture was fixed with interfragmentary lag screws and was further stabilized with a Variable Angle Locking Anterior Patella Plate and appropriately sized locking screws (Figure 1). The surgical site was copiously irrigated with sterile saline before the repair of the medial and lateral retinaculum of the knee with #2 Fiberwire suture. The deep tissues over the implant were approximated with #0 Ticron, and the surgical site was again irrigated with saline. Subcutaneous tissues were closed with a 2-0 polysorb suture and reinforced with a 3-0 V-lock suture. Finally, the wound was dressed with skin glue, and a sterile silver-impregnated dressing was placed over the incision. A tourniquet was used in conjunction with electrocautery, and blood loss was minimized to about 5cc. Following surgery, the patient was placed in a knee immobilizer and made to bear weight as tolerated on her right lower extremity with the aid of crutches. The patient was advised of standard post-operative instructions and wound care, showering as needed but avoiding excessive cleansing surrounding the surgical site until appropriate evaluation at a subsequent follow-up appointment.

The patient was seen for a two-week follow-up visit and was found to be healing expectedly and without concerns of wound infection or dehiscence. Her post-op dressing was removed, and Steri Strips were placed. The patient was again seen at the 4-week post-op mark for concerns of warmth, erythema, and clear drainage from her incision site. However, during her examination, these symptoms could not be corroborated during her visit, and she lacked signs of infection, including fevers, wound erythema, edema, effusion, or discharge. It was decided to treat the patient conservatively with close follow-up and ED follow-up should her symptoms acutely worsen.

**Figure 2:**
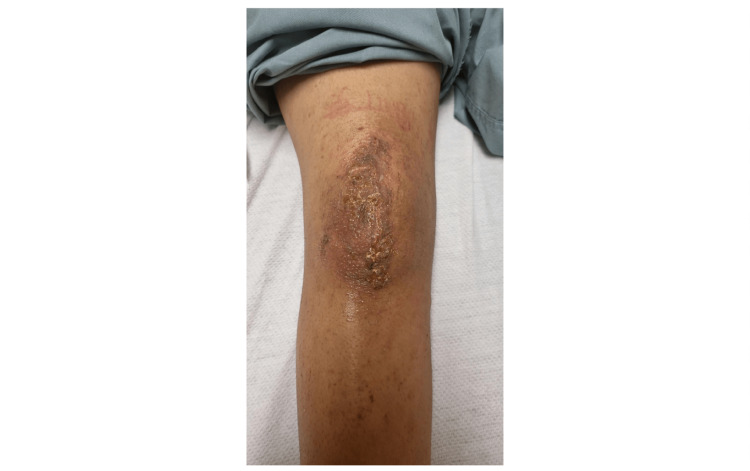
A five-week follow-up visit of the patient's right knee showcasing the surgical site infection with pathognomonic honey-crusted lesions indicative of impetigo

At the 5-week post-operative mark, the patient did present to the emergency department with complaints of peri-incisional erythema, swelling, and clear/yellow drainage from her incision. A clinical description of her incision at this time can be seen in Figure 2. At this time, the patient was afebrile. Laboratory examination during this time showed a WBC of 4.9 TH/uL (normal 3.8-10.8) with 64.8% neutrophils (normal 40.0-85%), C-Reactive Protein of 1.4mg/L (normal <8.0), and an Erythrocyte Sedimentation Rate of 42mm/h (normal < 20). A culture swab of the discharge was taken, and Gram staining was performed, which would later result in heavy growth and identification of Staphylococcus epidermidis, but the isolate was too fastidious for susceptibility studies. She was diagnosed with an impetigo surgical site infection at this time based on the clinical examination of her incision. Due to the superficial nature of the skin infection, the patient was discharged from the emergency room with oral amoxicillin 875mg-potassium clavulanate 125mg tablet twice daily as well as topical 2% mupirocin for 10 days with plans for weekly wound checks with orthopedics for resolution.

She was seen in the clinic about three days later. She had mild resolution of her symptoms but still presented with erythema and honey-crusted discharge. She was seen additionally one week later, this time with complaints of a systemic rash. Her infection site was improving but continued to demonstrate folliculitis with honey-colored discharge. Her prior oral and topical antibiotics were discontinued due to a presumed allergic reaction, and the patient was ordered oral trimethoprim/Sulfamethoxazole 800/160mg twice daily for ten days. Of note, the patient was also found to have a restricted range of motion at this visit and was scheduled for manipulation under anesthesia for right knee arthrofibrosis.

**Figure 3:**
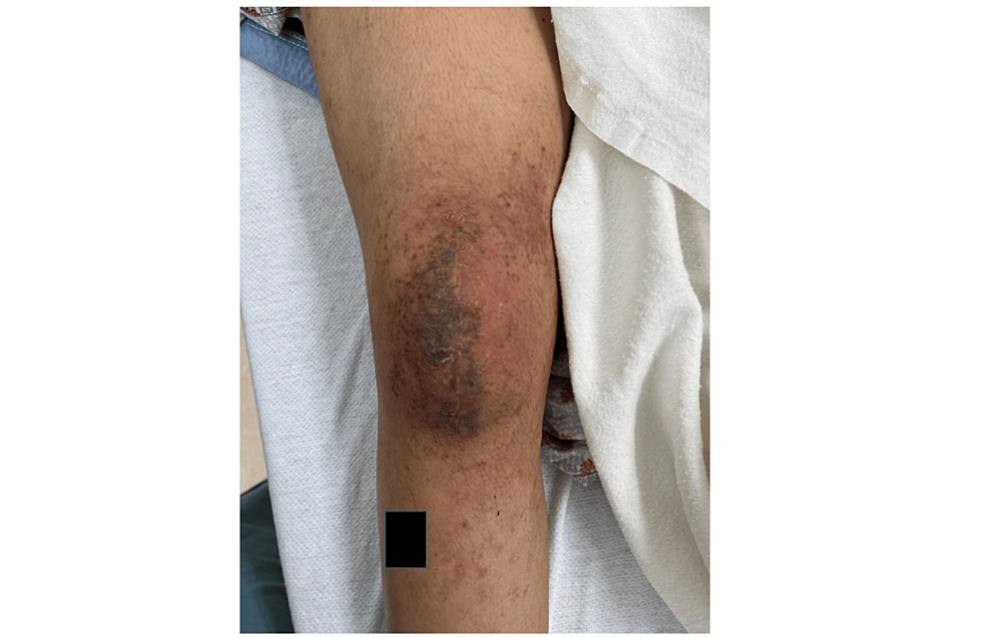
An eight-week follow-up photo of the patient's right knee surgical site infection following antibiotic treatment

The patient was brought back to the operating room about eight weeks following her initial procedure for manipulation of the right knee secondary to arthrofibrosis. She had been taking trimethoprim/sulfamethoxazole for ten days at this point. Pre-procedural examination of the skin of her right knee showed a resolution of honey-colored discharge with resolving erythema (Figure 3). At discharge for her procedure, her antibiotic was refilled for an additional 5 days. She was seen for a 2-week post-procedure check, and the resolution of her infection was noted.

## Discussion

We report a case of impetigo surgical site infection following a patellar fracture that required open reduction and external fixation in a 24-year-old female. Patellar fractures are most frequently reported in older adults, suggesting a possible osteoporotic/osteopenic component to the etiology. Rau et al. reported that women are at 1.5 times greater risk for patellar fractures, with the highest incidence in the 60 to 80-year-old age group, further supporting the osteoporotic theory [2]. Other sources report that males are at two times greater risk, demonstrating a contradiction in the literature on the genetic sex predominance for patellar fractures [1]. Patients in this age group are also more likely to have existing comorbidities, which also increases the rate of surgical complications. The mean time of occurrence for surgical site infections is between three and seven days, but many studies have reported a wide variance in the timeline [7]. The mean time of occurrence of surgical site infections after patellar fractures was found to vary significantly between studies, with studies reporting averages ranging from 5 days to 29.5 days with no genetic sex predominance [2,4,8]. This demonstrates that the presentation time of surgical site infections can vary depending on various factors, such as the extent and mechanism of injury, type of surgery, and reporting time, as some signs of infection may not be immediately noticed or reported. Our patient presented with initial concerns of infection on post-operative day (POD) 34, with antibiotic treatment initiated on POD55 as it was originally believed to be an allergic reaction.

In a four-year study analyzing the prevalence and risk factors associated with surgical site infection following patellar fracture surgery, Tan et al. reported an estimated prevalence of 2.1% of surgical site infections in 820 patellar fracture operations, with the most frequently identified microorganism being S. aureus [8]. Similarly, Forget et al. discovered that S. aureus accounted for 36.4% of all surgical site infections following orthopedic and trauma surgery cases in their prospective cohort, case-control study [9]. It was also noted that the biggest risk factors for developing an S. aureus surgical site infection were smoking and a National Nosocomial Infection Surveillance System (NNISS) score greater than one [9]. Additionally, diabetes, surgery duration >79.5 minutes, albumin <35 g/L, and being a carrier of S. aureus in the nasal passages of surgeons and patients have also been reported as risk factors for developing surgical site infections [8,10]. There was consensus agreement with the aforementioned risk factors and a general recommendation of a pre-operative nutrition and health screening, with subsequent pre-operative nutrition if the patient is malnourished [8]. It is important to note that pre-operative antibiotic showers and smoking cessation were found to be associated with a decreased risk of SSI. In contrast, prolonged operation time was associated with an increased risk of SSI, with the likely mechanism being related to an increased time for airborne dust and bacteria to seed into the wound [8,9]. Our patient denied a smoking history and had a total operation time of 138 minutes from incision to closure, demonstrating a prolonged operation consistent with the aforementioned risk factor.

Although impetigo is the most common skin infection in children two to five years of age, its incidence demonstrates a decline with increasing age [6,11]. Collectively, from a global epidemiological standpoint, Bowen et al. reported an impetigo infection rate of 4.9% for adults and 12.3% for children, with an estimated prevalence of 162 million affected children worldwide [12]. Impetigo is classified as either bullous or non-bullous, with the latter comprising 70% of cases [6]. Non-bullous impetigo typically begins around the mouth and nose as a red macule that can spread and progress to maculopapular lesions with thin-walled vesicles over a week [6,11]. It is important to note that these vesicles may rupture, resulting in the characteristic "honey-crusted lesions," which are highly contagious [11]. The non-bullous presentation is usually caused by Staphylococcus aureus infection; however, Streptococcus pyogenes is also a known culprit. S. aureus is a coagulase and catalase-positive, gram-positive cocci that grows in grape-like clusters and is found in normal skin flora, including the skin and mucous membranes, yet can cause potential infection by direct contact or fomite transmission [13]. Conversely, bullous impetigo primarily occurs in infants or immunocompromised patients and is characterized by the presence of large blisters that harbor infectious fluid and are typically localized in intertriginous areas [6]. The bullous presentation is typically due to the local production of exfoliative toxins A and B (ETA and ETB) by S. aureus bacteria.

Due to the inability of skin swabs to differentiate between bacterial infection and colonization, the first-line treatment for localized impetigo is selection of a topical treatment regimen based on factors including the likely bacterial pathogen, local bacterial resistance patterns, underlying disease, and affordability of medication [6,11]. As research detailing the most effective treatment for impetigo is limited, clinicians are advised to utilize their best judgment based on the aforementioned factors on a case-by-case basis [14]. Typically, topical mupirocin is initially utilized, while oral antibiotics such as amoxicillin/clavulanate, dicloxacillin, and cephalexin are reserved for patients who do not respond to treatment or have extensive disease involvement [6]. In these unresponsive patients, culture of the pus or bullous fluid may be beneficial in identifying the pathogen and determining anti-microbial specificity [6]. This approach should be considered in routine workup, as an increasing rate of resistance to commonly prescribed topical medications for impetigo has been noted [11].

## Conclusions

We report an impetigo infection in an adult female patient after patellar fixation, which is an uncharacteristic surgical site infection with no reported incidences in the literature. The patient tolerated the surgical procedure of interfragmentary lag screws and Variable Angle Locking Anterior Patella Plate and was capable of ambulation on the same day. The patient initially had an uncomplicated follow-up until she presented with early signs of infection on POD34 and continued to progress until POD55, when she was seen in the emergency department and treatment was initiated. The patient demonstrated complete resolution of the infection following treatment with trimethoprim/sulfamethoxazole and underwent manipulation under anesthesia due to arthrofibrosis of the knee, which was found to continue to improve at the last follow-up. This case report and literature review substantiate that patellar fracture repair is generally well-tolerated, but increased cognizance is needed for surgical site infections that may not be overtly obvious, especially uncharacteristic infections such as impetigo in an adult patient.
